# Effectiveness of Active Therapy-Based Training to Improve the Balance in Patients with Fibromyalgia: A Systematic Review with Meta-Analysis

**DOI:** 10.3390/jcm9113771

**Published:** 2020-11-22

**Authors:** María Del-Moral-García, Esteban Obrero-Gaitán, Daniel Rodríguez-Almagro, Manuel Rodríguez-Huguet, María Catalina Osuna-Pérez, Rafael Lomas-Vega

**Affiliations:** 1 Department of Health Sciences, Campus Las Lagunillas, University of Jaén, 23071 Jaén, Spain; mariadlmgr@gmail.com (M.D.-M.-G.); dralmagro4@gmail.com (D.R.-A.); mcosuna@ujaen.es (M.C.O.-P.); rlomas@ujaen.es (R.L.-V.); 2 Department of Nursery and Physiotherapy, University of Cádiz, 11009 Cádiz, Spain; manuel.rodriguez@uca.es; 3 Hospital de La Línea de la Concepción, 11300 Cádiz, Spain

**Keywords:** fibromyalgia, chronic fatigue syndrome, active therapy, postural balance, postural sensory organization, meta-analysis

## Abstract

Balance impairment is a frequent disorder in patients with fibromyalgia (FMS), increasing the risk of falls and decreasing physical function and quality of life. In recent years, the use of active therapy-based training (ATBT) has increased, with the aim of improving balance in women with FMS. Our study aimed to assess the effect of ATBT to improve different balance outcomes in subjects with FMS. A systematic review with meta-analysis was carried out. We searched PubMed Medline, SCOPUS, Web of Science, CINAHL, and PEDro (Physiotherapy Evidence Database) databases up to September 2020. We included randomized controlled trials (RCT) that assessed the balance in patients with FMS after ATBT and compared to other treatments or no intervention. In a random-effects model, the standardized mean difference (SMD) was used to calculate the effect size. Ten studies were included in the review providing data from 546 FMS patients with a mean age of 52.41 ± 2.90 years old (98% females). Our results showed a medium effect favors ATBT with respect to other therapies for monopedal static balance (SMD = 0.571; 95% CI = 0.305, 0.836; *p* < 0.001), dynamic balance (SMD = 0.618; 95% CI = 0.348, 0.888; *p* < 0.001), and functional balance (SMD = 0.409; 95% CI = 0.044, 0.774; *p* = 0.028). No statistically significant differences were found for balance on unstable support. The present meta-analysis showed moderate-quality evidence of a medium effect of ATBT to improve dynamic and functional balance and low-quality evidence of a medium effect to improve monopedal static balance with respect to other therapies or no intervention.

## 1. Introduction

Fibromyalgia Syndrome (FMS) is defined as a chronic disorder characterized by widespread and persistent non-inflammatory musculoskeletal pain that includes concomitant symptoms such as fatigue, insomnia, morning stiffness, depression, anxiety, and cognitive problems (forgetfulness, concentration difficulties, mental slowness, and memory and attention problems) [1]. Literature shows a worldwide FMS prevalence between 0.2% and 4.7%, and, specifically in women, between 2.4% and 6.8% [2]. On a social and personal level, FMS patients report difficulties in their family life and with their partner, because the typical FMS symptoms such as pain, anxiety, and depression can increase the perception that the women with FMS of not being able to meet the needs of their closest family bond (partner and/or children) [3], among which the deterioration of marital satisfaction stands out [4]. The social decline that people with FMS experience, coupled with a lack of support and understanding of their current health status, causes fibromyalgia to have a highly disabling impact on their lives [5,6]. Forty-four percent report they are fairly or totally dependent on a family member for household chores.

There are multiple evidence-based treatment guidelines for FMS, and all of them recommend standard symptomatic or pain pharmacological therapy (such as anti-epileptic drugs, anti-depressants, and muscle relaxants) and physical exercise in the form of aerobic, resistance, or flexibility training [7]. For example, the meta-analyses carried out based on the EULAR guideline, found that the only strong therapy-based recommendation was physical exercise [7]. Furthermore, initial management of the illness should involve patient education and focus on nonpharmacological therapies [8]. In case of non-response, further therapies should be tailored to the specific needs of the individual and may involve psychological therapies (for mood disorders and unhelpful coping strategies) [8], pharmacotherapy (for severe pain or sleep disturbance), and/or a multimodal rehabilitation program (for severe disability) [7].

Recent studies have found an alteration of postural control in FMS subjects [9,10,11]. FMS is also associated with an increased fall prevalence [12] and a greater awareness of their balance problems [10]. In addition, people with FMS have consistent objective sensory deficits as measured by posturography compared to healthy subjects [11]. Some studies have found a correlation between the impact of the FMS symptoms such as pain, muscle weakness, or stiffness can have on the ability to properly maintain balance [10,11]. Therefore, an improvement in balance can be expected to lead to a reduction in the impact of FMS.

The high frequency of balance disorders in patients with FMS and the resulting consequences, such as an increased falls risk, which can produce musculoskeletal damage added as bone fractures or disabling contusions [13] that reduce more the functional capacity of an individual with FMS, have led to a growing interest in the efficacy of exercise to improve balance and/or to reduce the risk of falls in subjects with FMS. Some reviews have tried to integrate the results reported in original research projects [14,15,16]. However, to the best of our knowledge, there are no meta-analyses analyzing the effect of this therapy on balance in FMS subjects. For this reason, the aim of this systematic review and meta-analysis was to search for the best evidence and to analyze the effects of active therapy-based training (ATBT) on balance in subjects with FMS.

## 2. Materials and Methods

### 2.1. Protocol Design

The Preferred Reporting Items for Systematic Reviews and Meta-Analyses (PRISMA) statement [17] was used to perform this systematic review with meta-analysis. The methodological protocol of this review was registered in PROSPERO International Prospective Register of Systematic Review (id number: CRD42020176976).

### 2.2. Data Sources and Search Strategy

Two authors (M.D.M.-G. and M.R.-H.) independently conducted a literature search strategy in PubMed Medline, SCOPUS, Web of Science, CINAHL, and PEDro (Physiotherapy Evidence Database) databases until September 2020. The authors also searched in the reference lists from retrieved full-text studies and reviews previously published. The PICOS tool proposed by the Cochrane Library [18] was used to identify potential studies in our search strategy: population (SFM); intervention (ATBT); comparison (no intervention or treatments different to active therapy); outcomes (balance classified in static, dynamic and functional); and study design (randomized controlled trial (RCT). According to the Medical Subject Headings (MeSH), we used the following terms as keywords in our bibliographic search strategy: “Fibromyalgia”, “postural balance”, and “exercise therapy”. A third author with expertise in bibliographic search (E.O.-G.) was consulted regarding any reservations related to the inclusion and combination of keywords and entry terms in the search strategy and the correct use of the Boolean operators “AND”/“OR” in each database. No language or publication date tags were set. Duplicated records were removed. Table 1 shows the bibliographic search strategy used in each database.

### 2.3. Study Selection and Inclusion Criteria

Two blind reviewers (D.R.-A. and M.C.O.-P.) independently screened the titles and abstracts of all references collected in the search strategy to identify potentially eligible studies. If at least one of the authors selected an article during the inclusion phase based on the title or abstracts, it was examined in detail. Differences that arose during full-text screening were resolved by a consultation with a third reviewer (R.L.-V.).

The inclusion criteria used were as follows: (1) Experimental studies, including RCT and RCT pilot studies; (2) studies that included patients diagnosed with FMS; (3) studies in which the intervention group received a treatment based on ATBT; (4) studies with a comparison group that received a different therapy to the intervention group or did not receive any therapy; (5) studies that assessed postural balance as an outcome measure; and (6) studies that provided data susceptible to be used in the quantitative synthesis. As exclusion criteria, we applied the following: (1) Cross-sectional and reviews studies; (2) RCT with intervention and control groups that were not exclusively composed of FMS patients, and (3) RCT studies that did not provide data to be used in the meta-analysis or transformed using validated procedures [18,19].

### 2.4. Data Extraction

Two authors (M.D.-M.-G. and D.R.-A.) collected the data from the selected studies using a standardized data-collection form in Microsoft Excel. A third author (E.O.-G.) was consulted to resolve any issues.

We extracted the following characteristics from the selected studies: research design, authorship, publication date, and sample size of each study. Concerning each comparison group (intervention or control group), we extracted the number of participants, age, gender, and body mass index (BMI). In addition, we collected the intervention and the control used in each study and their characteristics; balance was the outcome variable. We extracted the data (mean and its standard deviation) of the different tests and posturographic balance parameters used in these studies’ balance assessment. When mean or the standard deviation of the balance assessment was not available, we collected other statistical parameters, such as median, standard error, or inter-quartile range, susceptible to be transformed in mean and standard deviation and then included these in the meta-analysis [18,19].

### 2.5. Outcome Measures

The outcome measure was balance assessed with monopodal static balance tests, dynamic balance tests, functional balance tests, and balance assessment on an unstable support.

### 2.6. Quality Assessment

First, we assessed the risk of bias of the individual studies included in the review using the Cochrane Collaboration Risk of Bias Tool. This scale comprises seven items to provide information about the following bias: selection, performance, detection, attrition, reporting, and others. This tool labels risk as low, uncertain (when studies did not provide information about this), or high risk of bias.

Second, the overall quality of the evidence was assessed using the Grading of Recommendations Assessment, Development, and Evaluation (GRADE) system [20]. It provides information about the quality of the evidence in each meta-analysis taking into account the risk of bias of individual studies (previously assessed with the Cochrane Collaboration Risk of Bias Tool), inconsistency, indirectness, imprecision, and the risk of publication bias. We used the GRADE checklist by Meader et al. [21] to assess the inconsistency and imprecision. Inconsistency was evaluated through the heterogeneity [22] of the individual studies included (see statistical analysis), and imprecision was assessed by calculating the mean number of participants per study (high >300 participants, medium 300–100 participants, and low <100 participants), and the number of included studies (large >10 studies, moderate 5 to 10 studies, and small <5 studies) [20]. Details regarding the assessment of the risk of publication bias are shown in the statistical analysis section.

Two reviewers (M.D.M.-G. and D.R.-A.) independently assessed the risk of bias in individual studies and judged the overall quality of the evidence in each meta-analysis. The level of evidence of each meta-analysis was classified as: (1) high, showing robust findings; (2) medium, when our results may possibly change with new research; (3) low, showing a low level of confidence in the effect; and (4) very low, when any estimate of the effect is very uncertain. When a limiting factor was located, we downgraded the evidence by one level, and with the presence of several limitations, the overall quality was downgraded by two levels.

### 2.7. Statistical Analysis

We used the Comprehensive Meta-Analysis 3.3.070 software (Biostat, Englewood, NJ, USA) to perform the meta-analysis. Two authors were in charge of designing and developing the statistical analysis (E.O.-G. and R.L.-V.). The recommendations of Cooper et al. (2009) [23] were followed, and due to the heterogeneity found in the intervention group’s therapies and its characteristics, we chose the DerSimonian and Laird random-effects model to estimate the overall pooled effect with its 95% confidence interval (95% CI) to improve the generalization of the findings [24]. The pooled effect was estimated with the calculation of the Cohen’s standardized mean difference (SMD) [25], which may be interpreted at three effect strength levels: small (SMD = 0.2), medium (SMD = 0.5), and large (SMD > 0.8) [26]. The findings were displayed using the resulting forest plots [27]. The heterogeneity analysis was performed calculating the Q-test and the degree of inconsistency (I^2^) from Higgins that rates heterogeneity as low (<25%), medium (25–50%) and large (>50%) and its *p*-value calculation (*p* < 0.1 indicates large heterogeneity) [22,28]. The risk of publication bias was assessed with the asymmetry in the funnel plot [29], with the Egger test (*p* < 0.1 indicates the possible existence of publication bias risk) [30] and the adjusted pooled effect taking into account any possible publication bias calculated with the Trim and Fill method [31]. Related to publication bias, the quality level of evidence was not downgraded if the adjusted pooled effect, according to the Trim and Fill method, varied less than 10% with respect the original and raw pooled effect, although the funnel plot was slightly asymmetric.

### 2.8. Additional Analysis

A sensitivity analysis was performed with the leave-one-out method to assess each study’s contribution to the pooled effect in each meta-analysis [23].

## 3. Results

### 3.1. Study Selection

**Figure 1:**
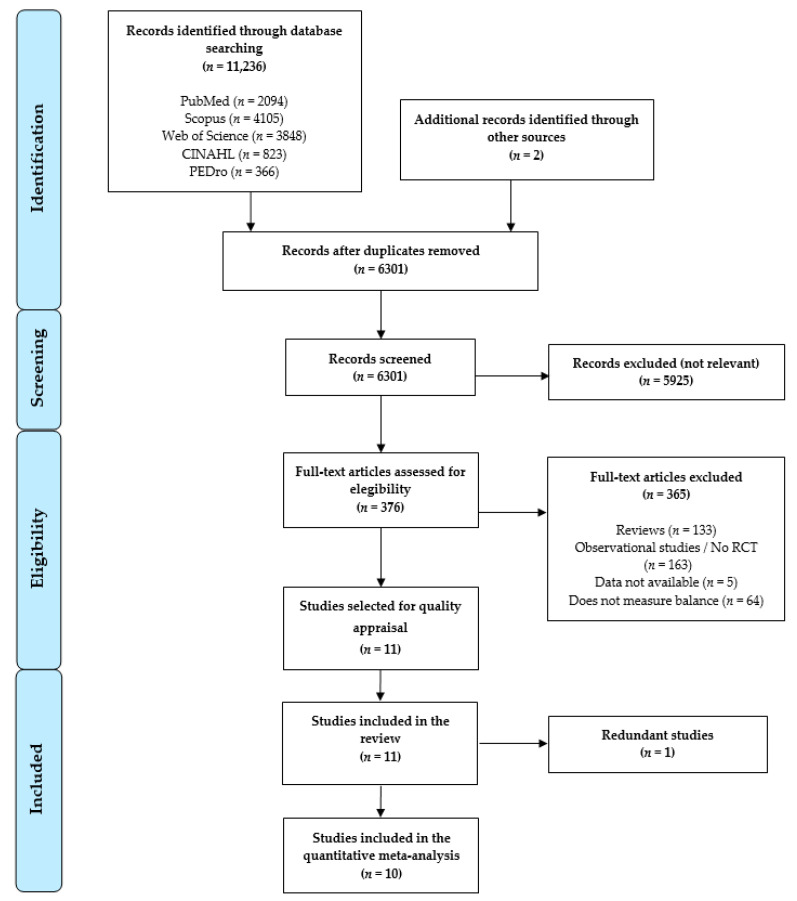
Preferred Reporting Items for Systematic Reviews and Meta-Analysis (PRISMA) flow chart.

The bibliographic search and the study selection process are displayed in the PRISMA flow chart (Figure 1). Initially, based on the search criteria, 11,236 references were retrieved from health databases, and 2 additional records were retrieved from other electronic resources. When duplicates were removed (*n* = 4937), 6301 references were analyzed by title/abstract, and 5724 were excluded for not being relevant. Five hundred seventy-seven full-text records were reviewed, and 365 were excluded for not meeting the inclusion criteria. In Figure 1 we show the number of references excluded along with the reasons. Finally, after the risk of bias assessment, 10 studies [32,33,34,35,36,37,38,39,40,41] were included in the present review.

### 3.2. Characteristics of the Studies Included in the Review

Ten studies [32,33,34,35,36,37,38,39,40,41] with 24 independent comparisons providing data for 546 FMS patients with a mean age of 52.41 ± 2.90 years old (98% females and BMI mean of 27.91 ± 1.83 kg/m^2^). The studies were undertaken in the following countries: Spain (7 studies) [32,33,34,36,37,39,41] and USA (3 studies) [35,38,40]. The intervention group was comprised of 270 subjects with FMS (51.81 ± 2.76 years old and 27.72 ± 1.85 kg/m^2^) who received an ATBT, such as core stability program [32], functional training [33], aquatic physical exercise therapy [34,36], Tai Chi [35], Yoga [40], physical exercise [37,38], or exergaming physical exercise [39,41]. The duration of the interventions proposed in each study lasted from 6 to 32 weeks. The control groups were made up of 276 participants with FMS with a mean age of 53.02 ± 3.03 years old and mean BMI of 28.11 ± 1.9 kg/m^2^ whose interventions were acupuncture [32], health education [35], usual care [40], or no therapy [32,33,34,36,37,38,39,41]. All studies included in this review were RCT and assessed the balance and its different domains: monopedal static [32,33,34,35,36], dynamic [32,33,35,39,41], and functional balance [32,37,38,39,40]. In addition, balance capability on an unstable support was assessed in 2 studies [39,40]. All balance assessments used in this meta-analysis were performed right after the intervention (immediate effect). Table 2 summarizes the main characteristics of the included studies.

### 3.3. Quality Assessment of the Studies Included in the Review

In Table 3, information is presented regarding the methodological quality evaluation and the risk of bias of the studies included in this review. No study was able to blind the type of ATBT or participants with FMS. Therefore, it is important to bear in mind that the risk of performance bias was high in all studies. In general, the overall quality of the included studies was moderate due to the possible presence in different studies of selection [33,34,35,36,38], performance, detection [33,34,35], and attrition bias [33,34,35]. Finally, five studies can be considered as higher quality than the others [32,37,39,40,41].

### 3.4. Meta-Analysis of the Immediate Effect of the ATBT in Monopedal Static Balance

**Figure 2:**
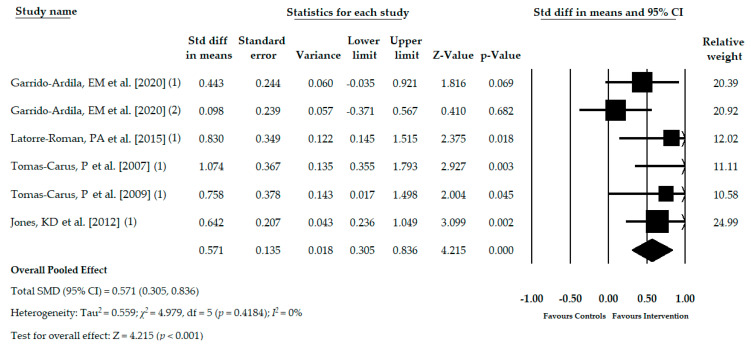
Forest plot of the meta-analysis of the immediate effect of the ATBT in monopedal static balance.

Five RCTs [32,33,34,35,36] with six independent comparisons provided data for 336 subjects with FMS (53.02 ± 2.32 years old, 97% female, and mean BMI 27.89 ± 1.80 kg/m^2^) in which the monopedal static balance was assessed using the one leg stance test. One hundred seventy-five participants (53.13 ± 2.43 years old) received an ATBT, such as core stability program [32], active-functional training [33], aquatic physical activity training [34,36], or Tai Chi [35]. In the control group, 162 individuals (52.9 ± 2.49 years old) received either acupuncture [32], health education [35], or no treatment [32,33,34,36]. The pooled effect (SMD = 0.571; 95% CI = 0.305, 0.836; *p* < 0.001) showed low-quality evidence of a medium effect of the ATBT in the improvement of monopedal static balance in patients with FMS in comparison with other therapies or no intervention (Table 4, Figure 2). The funnel plot appears slightly asymmetric, and the Egger test (*p* = 0.11) showed a possible risk of publication bias (Figure S1 in Supplementary Materials). The adjusted SMD (SMDadj = 0.477) effect, taking into account the possible risk of publication bias and calculated with the Trim and Fill method, showed a variation of 20% with respect to the original pooled effect. Heterogeneity was not present (I^2^ = 0%) and the number of participants per study was 56 showing a low level of precision. The sensitivity analysis (leave-one-out method) yielded pooled estimates that varied 18% when compared to the original pooled effect.

### 3.5. Meta-Analysis of the Immediate Effect of the ATBT in Dynamic Balance

**Figure 3:**
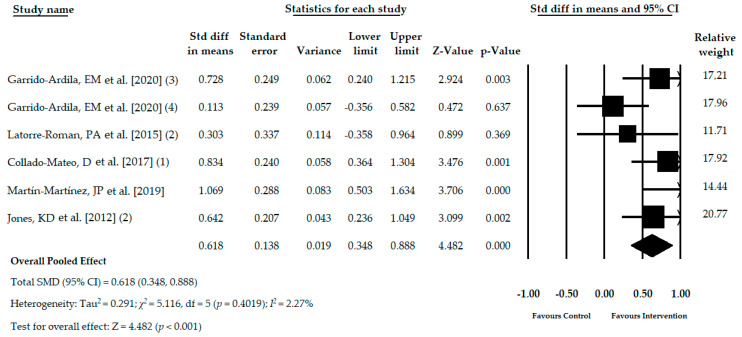
Forest plot of the meta-analysis of the immediate effect of the ATBT in dynamic balance.

Five studies [32,33,35,39,41] with six independent comparisons reported data of 403 participants with FMS (53.29 ± 2.69 years old, 98% female and BMI of 28.46 ± 2.35 kg/m^2^) in which the dynamic balance was assessed with the timed get up and go test. Two hundred and twelve participants received ATBT (53.91 ± 1.82 years old) using core stability program [32], active-functional training [33], exergame active therapy [39,41] or Tai Chi [35]. On the other hand, 191 participants (52.66 ± 3.41 years old) composed the control group receiving acupuncture [32], health education [35] or not treatment [32,33,39,41]. The duration of the active therapy intervention lasted from 6 weeks to 24 weeks. The pooled effect (SMD = 0.618; 95% CI = 0.348, 0.888; *p* <0.001) showed moderate-quality evidence of a medium effect of the ATBT in the improvement of dynamic balance in patients with FMS in comparison with other therapies or no intervention (Table 4, Figure 3). The symmetry found in the funnel plot, the Egger test *p* = 0.49, along with no variation in the Trim and Fill estimation, suggest our findings were without risk of publication bias (Figure S2 in Supplementary Materials). Heterogeneity was not present (I^2^ = 2.2%), and the level of precision was low (mean number of participants per study was 78.5). The sensitivity analysis estimated that the pooled effect varied 16% respect the original pooled effect.

### 3.6. Meta-Analysis of the Immediate Effect of the ATBT in Functional Balance

**Figure 4:**
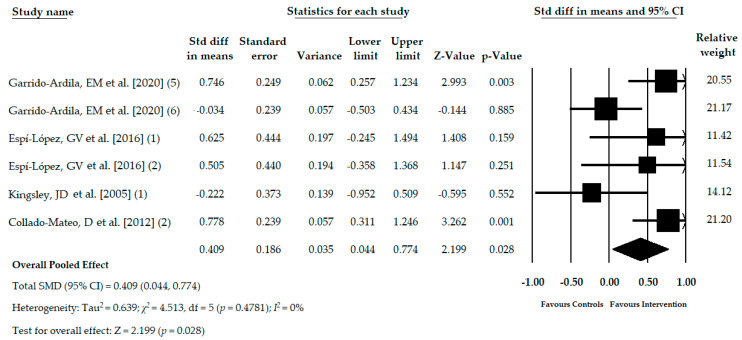
Forest plot of the meta-analysis of the immediate effect of the ATBT in functional balance.

Four studies [32,37,38,39] with six independent comparisons reported data for 288 participants with FMS (53.18 ± 3.90 years old, 99% female, and BMI mean of 27.74 ± 2.23 kg/m^2^) in which the functional balance was assessed with functional tests, such as Berg balance scale [32,37], the balance dimension of the continuous-scale physical functional performance [38], and functional balance reach test [39]. One hundred fifty-three subjects (52.30 ± 4.08 years old) received a core stability program [32], physical exercise with and without music therapy [37,38], yoga [40], and exergame active therapy [39]. On the other hand, the control groups comprised data of 135 women (54.05 ± 3.87 years old), of which no treatment was received in 4 studies [32,37,38,39] and in 1 study received acupuncture [32]. The pooled effect (SMD = 0.409; 95% CI = 0.044, 0.774; *p* = 0.028) showed moderate-quality evidence of a medium effect of ATBT in the improvement of functional balance in patients with FMS in comparison with other therapies or no intervention (Table 4, Figure 4). The risk of publication bias was not present as shown by the asymmetry in the funnel plot, the Egger test (*p* = 0.79), and no variation found in the adjusted SMD using the Trim and Fill method (Figure S3 in Supplementary Materials). Heterogeneity was nonexistent, and the precision level was low due to the mean number of participants per study (48). The pooled effect estimated by the one study removed method varied 32% with respect to the original SMD.

### 3.7. Meta-Analysis of the Immediate Effect of ATBT on Balance on an Unstable Support with Open and Closed Eyes

**Figure 5:**
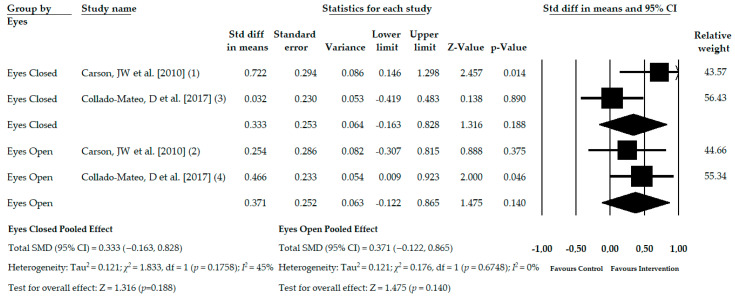
Forest plot of the meta-analysis of the immediate effect of the ATBT in the balance assessment on unstable support with eyes open and closed.

Two studies [39,40] with four independent comparisons provided data for 252 patients with FMS (53.33 ± 1.85 years old, 98% female, and BMI mean of 26.77 ± 1.13 kg/m^2^). The same number of subjects with FMS was examined on an unstable surface with eyes open and eyes closed (126 subjects with a mean age of 53.05 ± 1.90 years old in each condition). ATBT included yoga [40] and exergame active therapy [39], and control was usual care [40] or no intervention [39]. No statistically significant differences were found in the assessment of the bipedal balance on an unstable surface with eyes open (SMD = 0.371; 95% CI = −0.122, 0.865; *p* = 0.140) nor with eyes closed (SMD = 0.333; 95% CI = −0.163, 0.828; *p* = 0.188), both with a very low-quality evidence (Table 4, Figure 5). Heterogeneity was only present for the eyes-closed condition (I^2^ = 45%), and the mean number of participants per study was 63 for each condition, showing findings with a low level of precision. The risk of publication bias was unable to be calculated, although it is possible that was higher.

## 4. Discussion

Although the presence of balance alteration in subjects with FMS has previously been observed, as well as the health benefit that physical exercise implies for this population, a meta-analysis on the effects that physical exercise has on balance in persons with FMS has not been conducted to date. Thus, the present systematic review with meta-analysis aimed to analyze the effects of active therapy-based training (ATBT) on balance in persons with FMS. Although the benefits that physical exercise has in the management of patients with FMS has been reported previously, our results show the benefits that ATBT provides for balance in FMS patients.

The benefits of ATBT have been widely investigated in different populations. In the elderly population, it has been possible to observe that both monopedal static balance and dynamic balance can be improved through a combination of resistance and aerobic exercise and a training program where exergames and adapted physical activity would be combined [42]. Additionally, patients with multiple sclerosis found their balance and postural control improved, with a consequent decrease in falls risk, with flexibility and stretching exercises, as well as with a staircase exercise model [43]. Moreover, an exercise training program could also improve balance and gait ability and decrease fall rates, in both the short- and long-term, in patients with Parkinson’s disease [44].

In our study, it was possible to observe that ATBT not only improved monopedal static balance, but also dynamic balance, as well as functional balance. Both the severity of FMS symptoms and deficient physical function have been associated with postural control [10]. In this regard, exercise improves pain intensity and quality of life [45], which, jointly with the improvement that it causes in psychological function [45], could improve the severity perception of FMS symptoms [14]. It has also been possible to observe an enhancement in physical function due to exercise [45,46]. Previously, Jones et al. [10] suggested that postural control could be related to the severity of FM symptoms and an impaired physical function.

Daily life activities represent a challenge for persons with fibromyalgia as muscle strength represents a central component of the physical requirements for work and daily life activities [47]. It has been observed that reduced muscle strength may lead to a reduction in functional capacity [48]. Furthermore, lower limb muscle strength has been correlated with balance [49]: an association where age may have an important impact [49]. In this sense, not only has it been noted that persons with FMS present greater decreased lower limb muscle strength than healthy subjects [50,51], but also a functional capacity lower than women older than eighty years old [10]. Considering the above, it is possible to conclude the important role that physical activity plays in patients’ lives with FMS, along with the importance of improving their physical function [41] and, consequently, their balance [10], as our study results reflect.

On the other hand, there is evidence of the presence of a small fiber pathology (SFP) in patients with fibromyalgia [52]. Some studies have suggested that ankle joint proprioception and joint stability are more important to functional mobility in people with polyneuropathy [53], which could explain the poorer scores of subjects with fibromyalgia in tests of monopodal and functional balance.

Another aspect that could explain the improvement in balance obtained in the present systematic review is the positive effect of exercise therapy on cognitive function in patients with FMS [54]. The central nervous system employs anticipatory and compensatory postural adjustment (APA and CPA) to assure postural control regardless of the stability condition [55]. Therefore, cognitive capacity plays a major role in APA, and consequently, in postural control, as it affects predictive control and real-time processing of sensory information [56]. In this light, decreased processing speed and difficulties in executive function have been observed in patients with FMS [57], hindering successful APA, which could negatively impact functional independence. Thus, cognitive function improvement due to physical exercise [58,59,60] may lead to better APA, allowing improvement in postural control and functional balance.

Although our work shows a positive effect of ATBT on balance in patients with FMS, our results should be interpreted with caution because the improvement was observed in the short term. To date, there is not much evidence that ATBT can have a long-term effect. We believe that this may be due to the lack of specificity of the therapeutic programs implemented. Some studies have shown that patients with FMS could present a visual-vestibular problem more frequently [11,61,62] with a possible somatosensory dependence. Once it is known that the postural balance of patients with FMS can be addressed successfully, it would be advisable to implement specific balance exercises or vestibular rehabilitation programs to address the specific deficit, guiding the work more efficiently and seeking a more persistent benefit.

Some limitations of this study should be considered. First, the low number of studies included in this review can affect the generalization of our findings. Specifically, the low number of studies included in each balance condition meta-analysis may increase the risk of performance bias due to the impossibility to blind the participants in both groups and the risk of selection bias related to the randomization process in some studies. Second, the variety of different ATBT used in each study can make hinder the generalization of our findings favorable to a specific active therapy. The precision of our results may be affected due to the low number of studies included and the low number of participants per study in each meta-analysis. It is also important to note the possible limitation of the risk of publication bias in each balance condition due to the low number of studies selected and especially for monopedal and bipedal static balance. It is necessary to increase the number of clinical trials that assess the effectiveness of ATBT in subjects with FMS containing a large sample size to obtain robust findings with the aim to establish rehabilitation programs that use ATBT as a successful therapy.

## 5. Conclusions

Our findings show that a rehabilitation program using ATBT may be considered as an effective treatment to improve balance in persons with FMS. Moderate-quality evidence of a medium effect was found favoring ATBT with respect to other therapies or no intervention to improve dynamic and functional balance in subjects with FMS. Low-quality evidence of a medium effect for ATBT to improve the monopedal static balance was shown. However, no statistically significant differences were found in the assessment of bipedal balance on unstable support with eyes open and closed, with very low-quality evidence. This review shows the success of the active inclusion of subjects with FMS in active exercise programs to improve their balance. However, more research is needed to clarify essential aspects of ATBT, such as the duration of the intervention period and session frequency, and the type of ATBT indicated.

## Figures and Tables

**Table 1:** Search Strategy for each database.

Databases	Search Strategy
**PubMed Medline**	(fatigue syndrome, chronic[mh] OR fatigue syndrome, chronic[tiab] OR fibromyalgia[mh] OR fibromyalgia[tiab]) AND (exercise[mh] OR exercise[tiab] OR exercise therapy[mh] OR exercise therapy[tiab] OR physical exercise[tiab] OR physical activity[tiab] OR training[tiab])
**SCOPUS**	TITLE-ABS-KEY ((“chronic fatigue syndrome” OR “fibromyalgia” OR “fibromyalgia syndrome”) AND (“exercise” OR “exercise” OR “physical exercise” OR “physical activity” OR “exercise therapy” OR “training”))
**Web of Science**	TOPIC: (* chronic fatigue syndrome * OR * fibromyalgia * OR * fibromyalgia syndrome *) AND TOPIC: (* exercise * OR * physical exercise * OR * physical activity * OR * exercise therapy * OR * training *)
**CINAHL**	AB (“chronic fatigue syndrome” OR fibromyalgia) AND AB (exercise OR “exercise therapy” OR “physical activity” OR training)
**PEDro**	Fibromyalgia AND exercise

**Table 2:** Characteristics of the studies included in the meta-analysis.

		Experimental Group										Control Group					Outcome	
		Sample Characteristics						Intervention Characteristics				Sample Characteristics				Control Type	Balance Condition	Test
Author and Year	Country	*K*	*N*	*N*_e_	Mean Age	Mean BMI	% Fem	Type	Weeks	Ses /Week	Min	*N*_c_	Mean Age	Mean BMI	% Fem			
Garrido-Ardila, EM et al. (2020) [32]	Spain	6	103	36	56.1	-	100%	Core Stability	6	2	30	33	54.4	-	100%	NI	-Monopedal static -Dynamic -Functional	-OLST -TGUGT -BBS
												34	56.1	-	100%	Acupunc		
LaTorre-Román, PA et al. (2015) [33]	Spain	2	36	20	51.7	26.2	100%	Active-functional training	18	3	60	16	50.2	26.5	100%	NI	-Monopedal static -Dynamic	-OLST -TGUGT
Tomas-Carus, P et al. (2007) [34]	Spain	2	34	17	51	27	100%	Aquatic PA	12	3	60	17	51	27	100%	NI	-Monopedal static	-OLST
Jones, KD et al. (2012) [35]	USA	2	98	51	53.3	30.9	97%	Tai Chi	12	2	90	47	54.8	30.1	93%	Health education	-Monopedal static -Dynamic	-OLST -TGUGT
Tomas-Carus, P et al. (2009) [36]	Spain	1	30	15	50.7	28.8	100%	Aquatic PA	32	3	60	15	50.9	26.6	100%	NI	-Monopedal static	-OLST
Espí-López, GV et al. (2016) [37]	Spain	2	35	13	53.1	27	93%	PE + music	8	2	60	9	57.1	26.3	100%	NI	-Functional	-BBS
				13	51.2	26.3	93%	PE not music										
Kingsley, JD et al. (2005) [38]	USA	1	29	14	45	30.3	100%	PE	12	2	30	15	47	32	100%	NI	-Functional	-CS-PFP balance
Collado-Mateo, D et al. (2017) [39]	Spain	4	76	41	52.4	25.7	100%	Exerg. PE	8	2	60	35	52.5	27.7	100%	NI	-Functional -Dynamic -Unstable support	-FRT -TGUGT -CTSIB
Carson, JW et al. (2010) [40]	USA	2	50	22	51.4	-	100%	Yoga	8	1	-	28	55.8	28	100%	Usual Care	-Unstable support	-SCBT
Martín-Martínez, JP et al. (2019) [41]	Spain	1	55	28	54.04	27.36	100	Exerg. PE	24	2	60	27	53.4	28.8	100	NI	-Dynamic	-TGUGT

Abbreviations: K, number of comparisons; N, total sample size; Ne, experimental group sample size; BMI, body mass index; % fem, percentage of women; Ses, sessions; Min, minutes; OLST, one leg stance test; TGUGT, timed get up and go test; BBS, Berg balance scale; CS-PFP, continuous-scale physical functional performance; FRT, functional balance reach test; CTSIB, clinical test of sensory integration of balance; SCBT, sensory integration for balance Test; PA, physical activity; PE, physical exercise; Exerg, exergaming.

**Table 3:** Analysis of the Risk of Bias in the Included Studies.

	Selection Bias		Performance Bias	Detection Bias	Attrition Bias	Reporting Bias	Other Bias
Author and Year	Random Sequence Generation	Concealment of Randomization Sequence	Blinding of Participants	Blinding of Outcomes Assessors	Incomplete Outcome Data	Selective Reporting	Other, Ideally Prespecified
Garrido-Ardila, EM et al. (2020) [32]	-	-	+	-	-	-	-
LaTorre-Román, PA et al. (2015) [33]	-	?	+	?	?	-	-
Tomas-Carus, P et al. (2007) [34]	-	?	+	?	?	-	-
Jones, KD et al. (2012) [35]	-	+	+	+	?	-	-
Tomas-Carus, P et al. (2009) [36]	?	-	+	-	-	-	-
Espí-López, GV et al. (2016) [37]	-	-	+	-	-	-	-
Kingsley, JD et al. (2005) [38]	-	?	+	-	-	-	-
Collado-Mateo, D et al. (2017) [39]	-	-	+	-	-	-	-
Carson, JW et al. (2010) [40]	-	-	+	-	-	-	-
Martín-Martínez, JP et al. (2019) [41]	-	-	+	-	-	-	-

Abbreviations: “+” = high risk of bias, “-” = low risk of bias, “?” = inadequate data for the evaluation.

**Table 4:** Main findings in meta-analyses.

		Summary of Findings									Quality of Evidence (GRADE)					
		Pooled Effect					Het.	Publication Bias								
		*K*	*N*	*N*_s_	SMD	95% CI	I^2^ *(p)*	Funnel Plot Egger Test *(p)*	Trim and Fill		Risk of Bias	Incons.	Indirect.	Imprec.	Pub. Bias	Quality
									Adj SMD	% of Var						
Monopedal Static Balance																
**Overall**		6	336	56	0.571	0.305, 0.836	0% (0.4184)	Asym. (0.11)	0.477	20%	Medium	No	No	Yes	Likely	Low
**Dynamic Balance**																
**Overall**		6	403	78.5	0.618	0.348, 0.888	2.2% (0.4019)	Sym. (0.49)	0.618	0%	Medium	Not rel.	No	Yes	Unlikely	Moderate
**Functional Balance**																
**Overall**		6	288	48	0.409	0.044, 0.744	0% (0.4781)	Sym. (0.79)	0.409	0%	Medium	No	No	Yes	Unlikely	Moderate
**Bipedal Balance on an Unstable Support**																
**Eyes condition**	EO	2	126	63	0.371	−0.122, 0.865	0% (0.6748)	-	-	-	Medium	No	No	Yes	Likely	Very low
	EC	2	126	63	0.333	−0.163, 0.828	45% (0.1758)	-	-	-	Medium	Medium	No	Yes	Likely	Very low

Abbreviations: GRADE, grading of recommendations assessment development and evaluation; Het, heterogeneity; K, number of comparisons; N, number of participants in each meta-analysis; N_s_, mean of participants per study; SMD, Cohen standardized mean difference; CI, confidence interval; I^2^, Higgins degree of inconsistency; *p*, *p*-value; Adj, adjusted; % var, percentage of variation; Incons, inconsistency; Indirect, indirectness; Imprec, imprecision; Pub. Bias, publication bias; Sym, symmetric; Asym, asymmetric; EO, eyes open; EC, eyes closed.

## Supplementary Materials

The following are available online at https://www.mdpi.com/2077-0383/9/11/3771/s1, Figure S1: Funnel plot of the meta-analysis for monopedal static balance; Figure S2: Funnel plot of the meta-analysis for dynamic balance; Figure S3: Funnel plot of the meta-analysis for functional balance.
