# Supplementary figures and images for: Effectiveness of Active Therapy-Based Training to Improve the Balance in Patients with Fibromyalgia: A Systematic Review with Meta-Analysis
Source: J Clin Med. 2020 Nov 22;9(11):3771. doi: 10.3390/jcm9113771 (PMC7700277; doi:10.3390/jcm9113771)

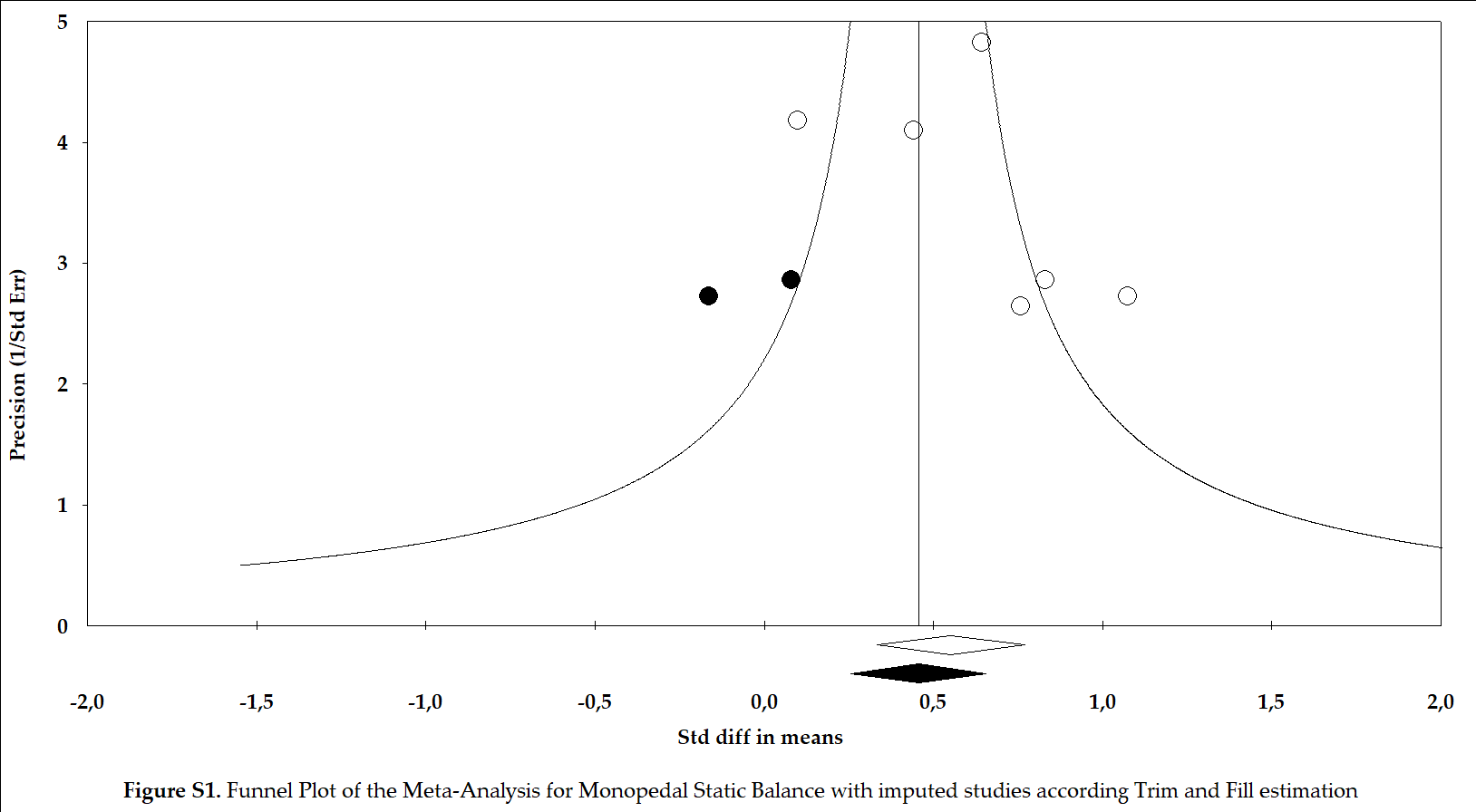

Supplement: Supplementary file 1 [file jcm-09-03771-s001.zip › jcm-1005636 Suplementary files/Figure S1. Funnel plot for monopedal static balance.tif]

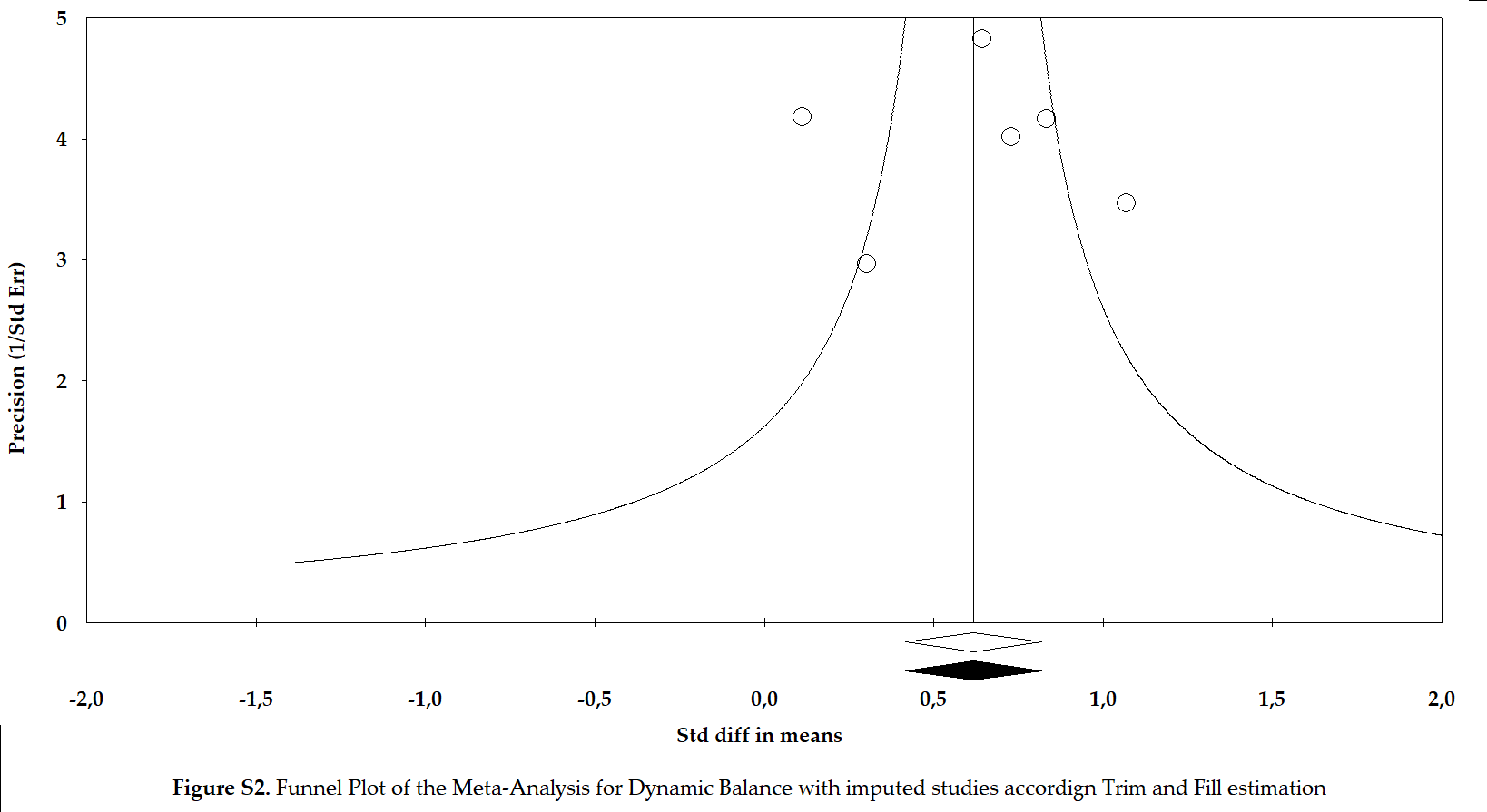

Supplement: Supplementary file 1 [file jcm-09-03771-s001.zip › jcm-1005636 Suplementary files/Figure S2. Funnel plot for dynamic balance.tif]

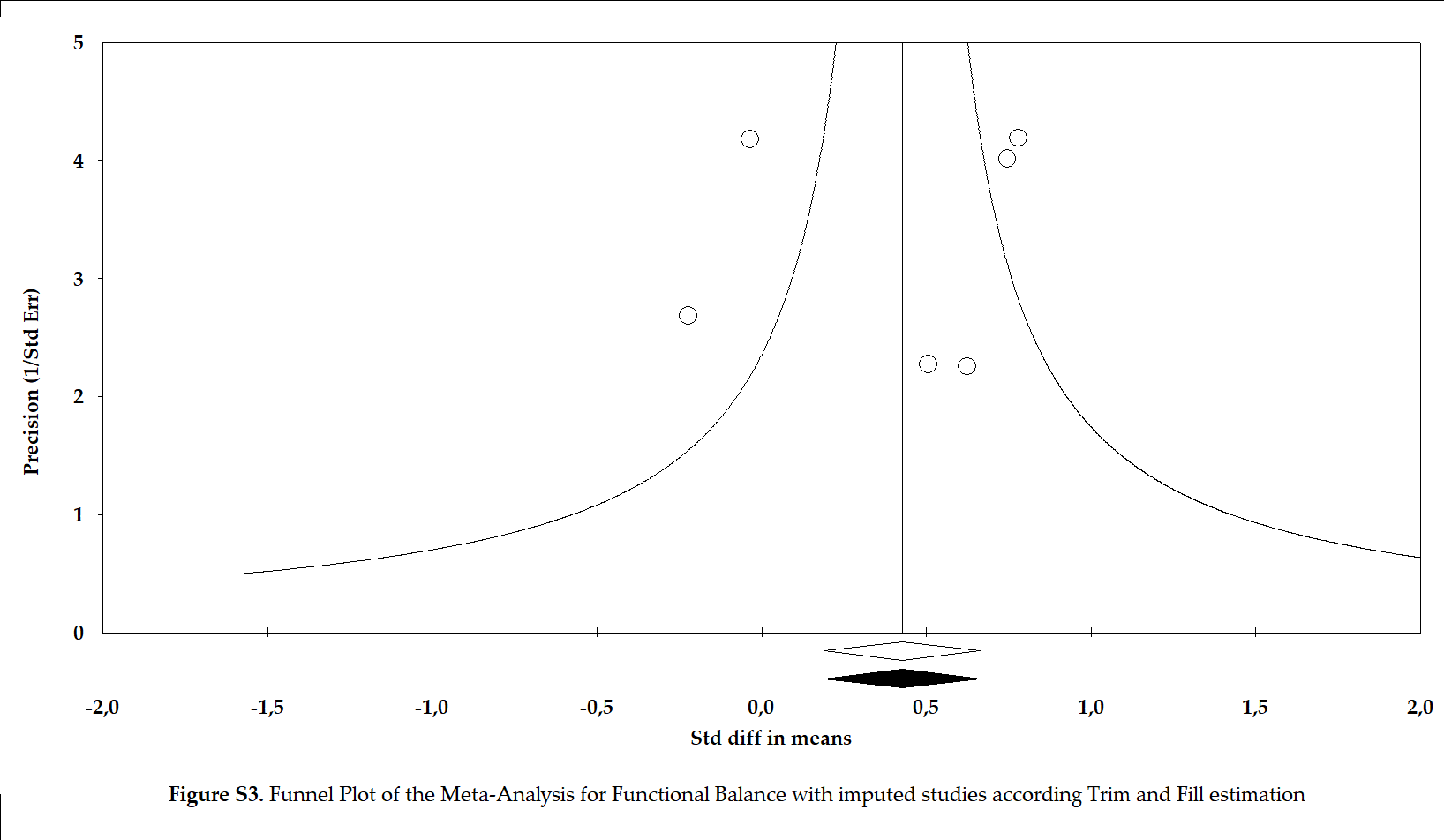

Supplement: Supplementary file 1 [file jcm-09-03771-s001.zip › jcm-1005636 Suplementary files/Figure S3. Funnel plot for functional balance.tif]
